# Electrochemical deposition of highly hydrophobic perfluorinated polyaniline film for biosensor applications†

**DOI:** 10.1039/d1ra02325j

**Published:** 2021-05-25

**Authors:** Elena Tomšík, Panagiotis Dallas, Ivana Šeděnková, Jan Svoboda, Martin Hrubý

**Affiliations:** Institute of Macromolecular Chemistry AS CR 162 06 Prague 6 Czech Republic tomsik@imc.cas.cz; Institute of Nanoscience and Nanotechnology, NCSR Demokritos Patriarhou Grigoriou & Neapoleos 15310 Greece

## Abstract

Highly hydrophobic perfluorinated polyaniline thin films with water contact angle of ∼140° and low internal resistance properties are prepared through electrochemical polymerization. UV-visible spectroscopy demonstrates a gradual evolution of the polaron band which indicates the electronic conductive properties of the polymers. Simultaneous possession of the water-repelling property and electron conductivity for superhydrophobic perfluorinated polyaniline leads to a unique polymer that is suitable as a solid contact in ion-selective electrodes for *in situ* monitoring of pH changes during early stages of inflammation and septic shock. The superhydrophobic properties should suppress interactions with interfering salts and proteins, and the sensitivity towards protons could be monitored by measuring the phase boundary potential, which depends on the H^+^ concentration. The potentiometric measurements demonstrate a fast response with a slope of 44.4 ± 0.2 mV per unit pH. The presence of interfering ions and/or human serum albumin does not have any significant effect on the performance of the perfluorinated film. Moreover, it is demonstrated that the response of the perfluorinated film is reversible within the biomedically relevant pH range from 4.0 to 8.5, and stable over time.

## Abstract 2

Simultaneous possession of the water-repelling property and electron conductivity for superhydrophobic perfluorinated polyaniline leads to a unique polymer that is suitable for *in situ* monitoring of pH changes during early stages of inflammation and septic shock.

## Graphical abstract

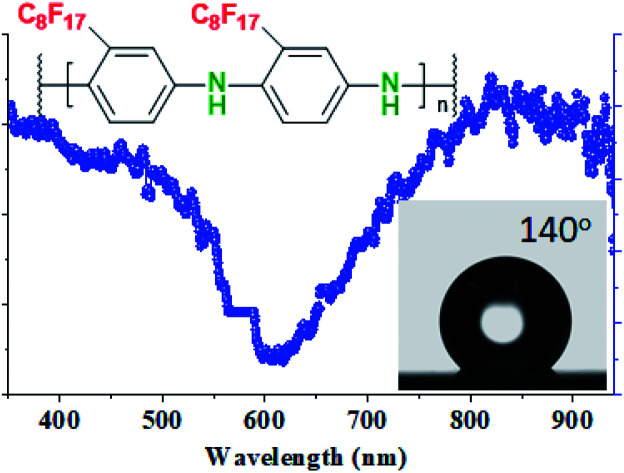

## Introduction

Electrically conducting polymers (ECPs) such as polyaniline (PANI), polypyrrole (PPY), and poly(3,4-ethylenedioxythiophene) (PEDOT)^1–3^ are suitable as solid contacts for the development of solid-contact ion-selective electrodes (SC ISEs).^4^ The criteria for solid contact materials are as follows: the p-doped ECP has to have a low oxidation potential, which translates to being in its conducting form, and possess a hydrophobic interface that will prevent an aqueous layer from forming between the ion-selective membrane and the solid contact (SC).^4^ The layer should also be resistant to interference from system components other than the measured one. Generally, when the ECP is in its oxidized form, it induces a large charge density on the polymeric backbone, which can be compensated by the incorporation of charge-balancing counter ions. Additionally, increased charge density commonly leads to an increase in the film’s hydrophilicity,^4^ and thus allows the formation of a water layer at the surface of ECPs. To eliminate the unwanted presence of water, the synthesis of hydrophobic ECPs is usually performed with the incorporation of hydrophobic ions in one step during the polymerization process.^4,5^ Another method is to use derivatives of highly hydrophobic monomers, for example, PEDOT-C_14_, as recently reported by Lindner *et al.*^6,7^ The authors used PEDOT-C_14_ as an ion-to-electron transducer and proved that the film possesses superhydrophobic properties with a water contact angle of 136 ± 5°. Another source of hydrophobic properties is to use side chains with fluorine atoms in their structure. Recently, it was demonstrated that the incorporation of perfluorinated electron-withdrawing hydrocarbon moieties into the monomer leads to unique characteristics in the oligomers, such as fluorescence, that can be tuned at will.^8,9^ The versatile liquid–liquid interface polymerization method was employed for the synthesis of light-emitting oligomers and hydrophobic polyaniline films that can be readily removed from the interface.^8^ However, the electrochemical polymerization, deposition, and characterization of this specific monomer have not yet been reported in the literature. With respect to the above, we proceeded to use 3-heptadecafluorooctyl aniline as a monomer for electrochemical deposition of a hydrophobic film and electrochemically characterize it for potential application as a solid contact. For practical applications, a simple and highly efficient method for the formation and deposition of superhydrophobic films is necessary. The superhydrophobicity of the perfluorinated chains is an advantage for pH sensors that should work in the biological milieu – they repel not only water, salts, and hydrophilic molecules, but (unlike the C_14_ chains) also hydrophobic moieties (the “Teflon” effect) such as hydrophobic blood protein domains or fatty acids, thus reducing unwanted interference from such molecules in H^+^ concentration sensing.

The sensing of pH in real-time in the biological milieu is of tremendous importance. It can be used for local pH sensing to detect inflammation around smart implants (typically accompanied by a local pH value drop),^10–12^ for early detection of septic shock, which is critical for the survival chance of the patient (one of the earliest symptoms is acidosis in urine/blood),^13–15^ or for gastric pH monitoring relevant to pathologies such as gastric ulcers. Such sensors should be miniaturized (potentiometric sensors are very advantageous here) and should not show interference with body fluid components other than H^+^ ions (proteins, salts, *etc.*).

In this work, we demonstrate that a pH-sensing 3-heptadecafluorooctyl aniline polymer can be readily electrochemically deposited on an electrode by the galvanostatic method with controlled mass loading, and with the formation of a uniform superhydrophobic polymer film in its oxidized state. Moreover, potentiometric measurements on such films show a Nernstian response in a broad range of pH. The presence of interfering ions (artificial urine) and/or human serum albumin in the studied analyte does not influence the sensitivity of the perfluorinated polyaniline films towards H^+^.

## Results and discussion

### Polymer film deposition and structural characterization

**Fig. 1:**
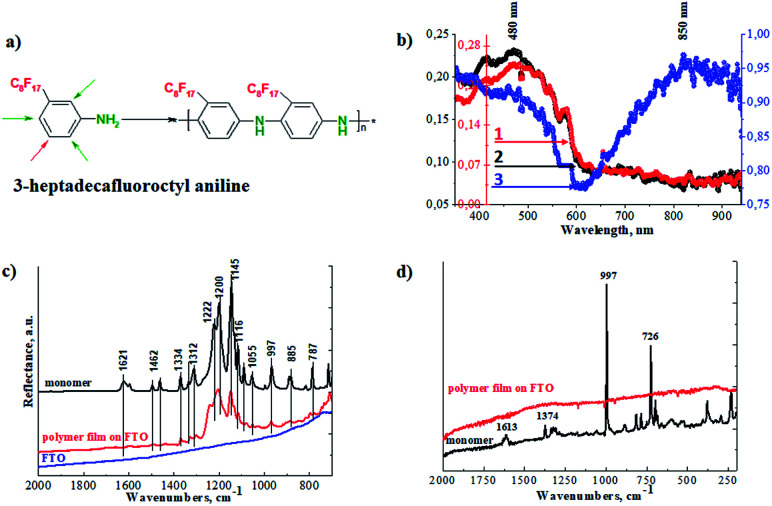
(a) The 3-perfluorooctyl aniline monomer and the electropolymerized polymer. (b) UV-visible spectra of perfluorinated films electrochemically deposited on FTO electrodes for various times: (1) 1000 s, (2) 2000 s and (3) 4000 s; (c) FTIR ATR spectra of the FTO electrode, the monomer and the polymer deposited on FTO; and (d) Raman spectra of the monomer and the polymer film recorded at laser wavelength *λ* = 785 nm.

Superhydrophobic perfluorinated polyaniline films were deposited on FTO electrodes through electrochemical polymerization (Fig. 1a). The electropolymerization was carried out galvanostatically by applying a 1 mA current for 4000 s. To find the optimal electropolymerization time, the film deposition was performed for 500 s, 1000 s, 2000 s, and 4000 s (a plot of mass loading *vs.* duration of deposition is presented in the ESI in Fig. S1†). It is obvious that after 1500 s, the increase in mass loading is suppressed and reaches a plateau. After careful evaluation of the experimental data, we decided to use 4000 s as the duration of the polymer film deposition, with a detailed explanation to be presented below. To identify the chemical structure of the polymer films, FTIR, Raman, and UV-vis spectroscopy was applied. As a first step, we recorded the UV-visible spectra, and the results are presented in Fig. 1b. The spectra of the polymer films obtained after different electropolymerization durations evolve: with increasing duration, the absorption maximums are shifted towards a longer wavelength and resemble the polaron band of polyaniline.^17–20^ This is an important observation and signals the formation of the conductive form of the material. The low resistance of the deposited film is confirmed below by electrochemical impedance spectroscopy (EIS).

In our case we observed the formation of a polaron band at 850 nm (Fig. 1b). This is the lowest energy gap for a polaron band recorded so far for perfluorinated polyaniline. Önal *et al.* published results of electrochemical polymerization of fluoro-substituted anilines, however, no polaron bands were observed by his group.^21^ Also, Montilla *et al.* reported the co-polymerization of fluorinated polyaniline. The formation of polaron bands was recorded at 770 nm.^22^

In the second step, we employed FTIR spectroscopy, which can provide information on the oxidation state of the polymer films, and the results are shown in Fig. 1c. The most pronounced bands in the spectrum of perfluorinated aniline are found between 1260 cm^−1^ and 1100 cm^−1^ and are assigned to the vibration of the fluorinated alkyl chain on the aniline ring. These bands are preserved in the spectrum of the electrochemically prepared polymer and their positions are shifted to higher wavenumbers. These sharp bands are broadened in the spectrum of the polymer. The less intense bands associated with the vibrations of the 1,3-disubstituted benzene ring are suppressed as the vibrations of the benzene and/or quinone rings are restricted in the polymer structure.^23^ The intensity of the vibrations associated with the benzenoid units in the polymer is very small compared to the monomer. The detailed spectra of the monomer and polymer (in the range from 1200 cm^−1^ to 1700 cm^−1^) are presented in the ESI in Fig. S2.†

Raman spectra were recorded and are presented in Fig. 1d. The Raman spectrum of the monomer exhibits two intense bands. The first one at 997 cm^−1^ is linked to the in-plane deformations of the 1,3-disubstituted benzene ring, and the second one at 726 cm^−1^ is linked with the C–F deformation vibration of the perfluorinated alkyl chain. After the electrochemical oxidation of the monomer, the strong fluorescence of the product makes the measurement of the Raman spectrum impossible. Based on this result, we can conclude that the monomer was successfully electropolymerized.

The presence of strong photoluminescence in perfluorinated aniline oligomers synthesized by liquid–liquid interface polymerization has already been reported in the literature by Dallas *et al.*^8,9^

**Fig. 2:**
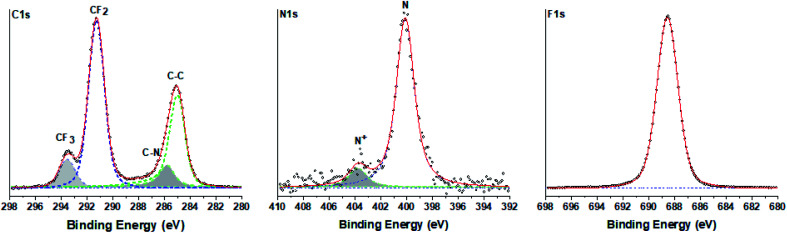
High-resolution C 1s, N 1s and F 1s X-ray photoelectron spectra of the electrochemically deposited film.

To determine the chemical composition of the perfluorinated polyaniline, the electrochemically deposited film was rinsed with distilled water and dried in an oven overnight and then analyzed by high-resolution X-ray photoelectron spectroscopy (XPS). The results of the measurements are presented in Fig. 2 for C 1s, N 1s, and F 1s. The optimum fitting of the C 1s signal consists of two major peaks (at 285.0 eV and at 291.3 eV), and two minor peaks (at 285.8 eV and at 293.6 eV). The first major peak at 285.0 eV corresponds to aromatic carbon, and the following minor peak at 285.8 eV is assigned to carbon bound to nitrogen. The second major peak at 291.3 eV corresponds unambiguously to fluorinated carbon (–CF_2_–) and the peak at 293.6 eV corresponds to –CF_3_ carbon. It is interesting to analyze the N 1s signal. The peak could be deconvoluted into one main peak at 400.1 eV that corresponds to unprotonated nitrogen and one minor peak at 403.8 eV assigned to oxidized nitrogen.

The presence of oxidized nitrogen will play a crucial role in the electrochemical performance of the deposited film. This fact is analyzed by electrochemical impedance spectroscopy and presented below. The survey spectrum of the perfluorinated polyaniline film is shown in the ESI in Fig. S3.†

**Fig. 3:**
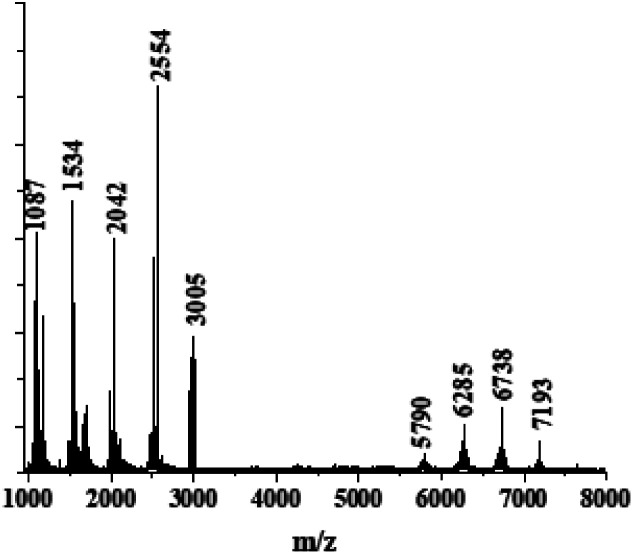
MALDI-TOF measurements on polymer powder.

To determine the number of monomer units in the polymer chains, MALDI-TOF analysis in the negative ionization mode was used, and the results are presented in Fig. 3. We observed a bimodal distribution of molecular weight. The highest peak corresponds to the pentamer with *m*/*z* = 2554. Oligomers with longer chains were detected as well, corresponding to up to 14 monomer units in the chain with *m*/*z* = 7193. However, the intensity of the last peak is much smaller. We suggest that higher intensities are observed for the shorter chains because they are easier to evaporate, and/or we might observe partial degradation of the longer chains by the laser beam.

**Fig. 4:**
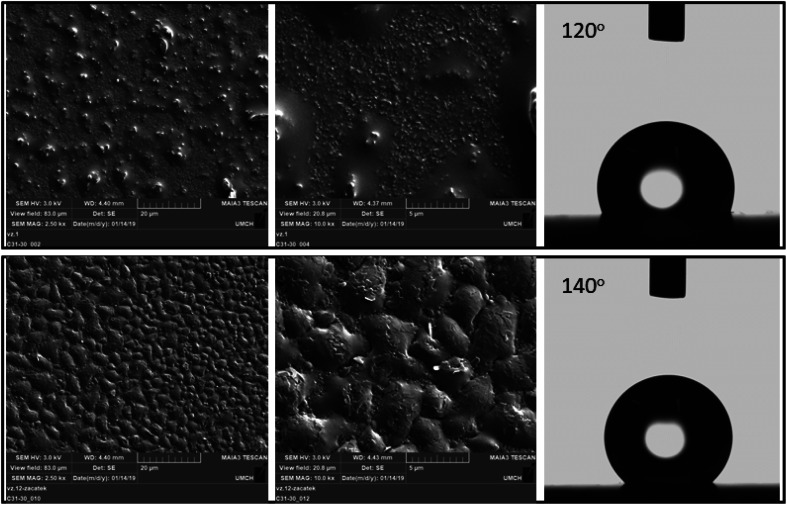
SEM images of perfluorinated films with different water contact angles. The top images represent the perfluorinated polyaniline film deposited for 2000 s, and the bottom images represent the perfluorinated polyaniline film deposited for 4000 s.

As shown by the scanning electron microscopy images in Fig. 4, the surface of the polymer film changes with the duration of deposition. Specifically, upon increasing the deposition duration, the roughness and the homogeneity of the surface increased.

Top views of the deposited perfluorinated polyaniline films are obtained by scanning electron microscopy (Fig. 4). While perfluorinated polyaniline synthesized through interfacial polymerization grows in flakes or fibers, the electropolymerized material forms a uniform thin film without the presence of any distinctive morphologies, and the images reveal that a compact structure is obtained. With increasing deposition time, the roughness of the film is increased and this manifests in the very high water contact angle of 140 ± 5° (*n* = 10). To the best of our knowledge, this is the highest value to date for a water-repelling conductive polymer. We must say that another fluorine-containing polymer was investigated and its water contact angle was 116°, as reported by Tsarevsky *et al.*^24^ A high water contact angle for a non-fluorinated polymer was measured by E. Lindner *et al.* for a hydrophobic derivative of poly(3,4-ethylenedioxythiophene), and it was 136 ± 5°.^6^

The static water contact angle measurements reveal that by changing the deposition duration we can tune the water contact angle of the perfluorinated films from 120° to 140°. Based on the SEM images, we conclude that with increasing deposition duration, the roughness of the perfluorinated polyaniline increases and contributes to the increase in the water contact angle.

### Electrochemical characterization

**Fig. 5:**
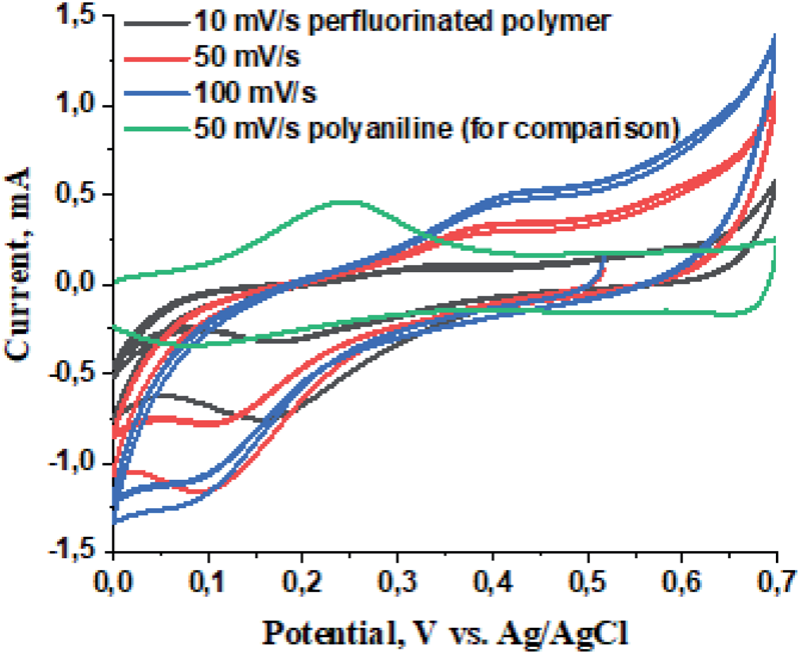
Cyclic voltammetry of the perfluorinated polymer at different scan rates and polyaniline at 50 mV s^−1^ for comparison.

**Fig. 6:**
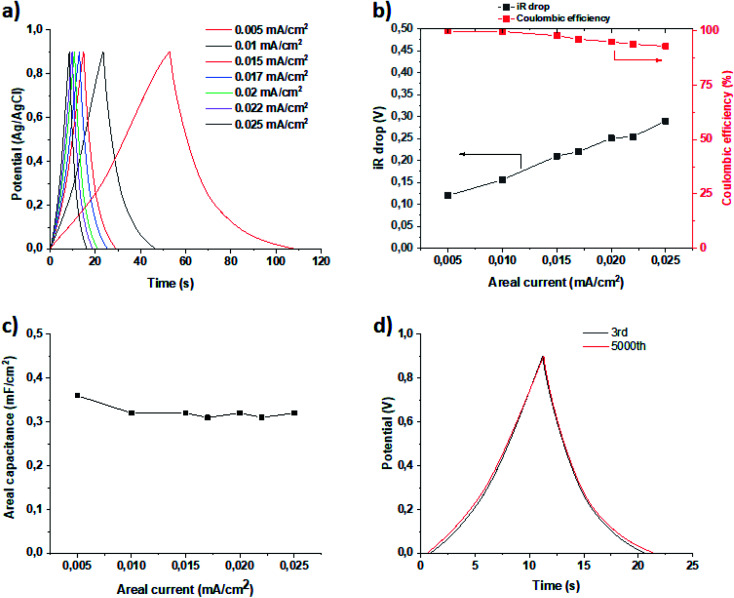
GCD measurements (a), *iR* drop and coulombic efficiency (b), plot of areal capacitance *vs.* areal current density (c), and stability test (d).

**Fig. 7:**
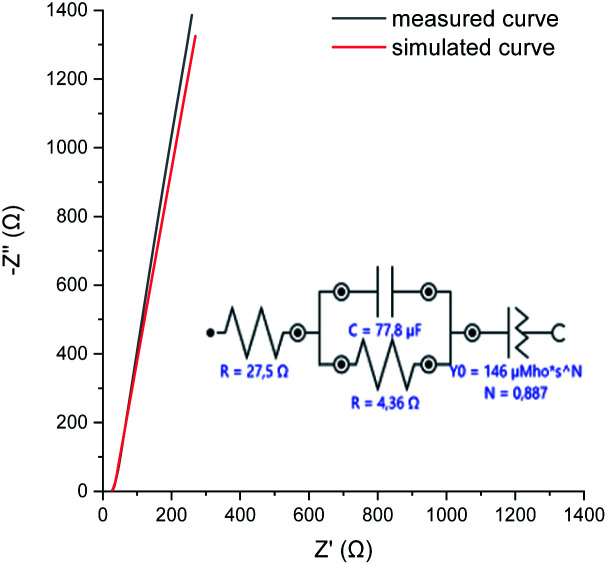
EIS measured at OCP, and the simulation model used to describe the physical processes taking place in the perfluorinated polyaniline film.

To evaluate the electrochemical properties of the perfluorinated polyaniline film, cyclic voltammetry was carried out in a three-electrode cell configuration (Fig. 5), galvanostatic charge/discharge measurements are presented in Fig. 6, and electrochemical impedance spectroscopy was recorded at open circuit potential (0.4 V *vs.* Ag/AgCl) (Fig. 7).

The cyclic voltammetry was conducted at three scan rates: 10, 50, and 100 mV s^−1^. We included the cyclic voltammetry of polyaniline (without the presence of perfluorinated chains) at a scan rate of 50 mV s^−1^ for comparison.^17^ From the voltammograms we conclude that the oxidation potential is located at 0.4 V *vs.* the Ag/AgCl electrode and the reduction potential is situated at 0.15 V *vs.* Ag/AgCl. The higher oxidation potential for perfluorinated polyaniline compared to polyaniline (0.2 V *vs.* Ag/AgCl) can be explained by the presence of the electron-withdrawing perfluoroalkyl chain in the *meta*-position.^17^ The difference between the oxidation and reduction potentials is ∼300 mV, which means that the electrochemical process is quasi-reversible. This fact is also proved by the shifting of both potentials with increasing scan rate.

Galvanostatic charge/discharge (GCD) measurements were employed to determine the capacitance of the deposited film, and the study was done with a three-electrode cell configuration. The results of the measurements are presented in Fig. 6. The areal capacitance was calculated according to eqn (1)

1 *C* = (*I* × *τ*)/(*V* − *iR*)

where *I* is the areal current density (A cm^−2^), *τ* is the discharge time, and (*V* − *iR*) is the measured voltage without *iR* drop.

It is worth noting that the perfluorinated polyaniline film possesses a capacitance of 0.36 mF cm^−2^, and that this capacitance does not change with areal current density (Fig. 6c). Of course, the value of areal capacitance is much smaller compared to the literature data recorded for polyaniline.^25–30^ This fact could be explained by the presence of large perfluorooctyl side chain groups in the *meta*-position. The presence of these bulky chains also means that not all of the surface is in contact with the electrolyte. Nevertheless, simultaneous possession of a WCA of 140° and relatively high areal capacitance makes the perfluorinated polyaniline film an ideal candidate for solid contacts.

With further analysis of the GCD curves, we can see that the coulombic efficiency is almost 100%, as presented in Fig. 6b. Even after increasing the areal current density 5-fold, the coulombic efficiency only drops down to 93%. This means that the charge and discharge times are equal and do not depend on the areal current density. Another important factor is the *iR* drop, which is small, as shown in Fig. 6b.

It is worth noting that the stability of the perfluorinated film was tested by GCD at 0.02 mA cm^−2^ current density (Fig. 6d). After 5000 cycles, the electrochemical performance of the film did not change, which provides proof that the film is stable under the measurement conditions.

The electrochemical impedance spectroscopy (EIS) measured at open circuit potential shows that the resistivity of the deposited perfluorinated polyaniline is low, ∼4.4 Ω (corresponding to 12.3 Ω cm^2^), which means that the film is conductive in its oxidized form (Fig. 7).

EIS is the method of choice for characterizing the electrical behavior of systems in which the overall system behavior is determined by strongly coupled processes, each proceeding at a different rate. The EIS data are analyzed by fitting to an equivalent electrical circuit model, and the model for our measurements is presented in Fig. 7. The fitting parameters are shown next to each element. In our system, we observed double layer capacitance (areal capacitance), and the fitted value is higher compared to the results from GCD. The difference is explained by the fact that in one method, a direct current is applied, and in the other one, an alternating current is used. The last element in the fitting model is the constant phase element. Such an element is used when the substrate surface is rough, as was suggested before.^31,32^ The results of the simulation are presented in Fig. 7 as a red line, and as can be seen, they closely follow the measured data.

### Potentiometric measurements under different conditions

**Fig. 8:**
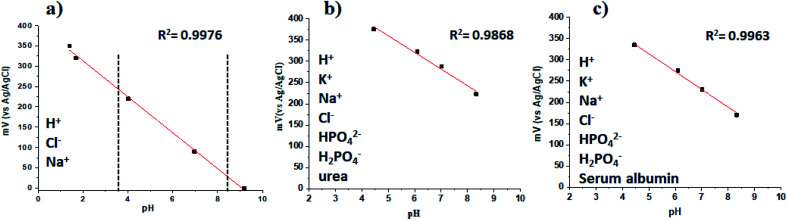
Potentiometric measurements of perfluorinated films under various conditions: (a) different concentrations of protons, (b) protons with interfering ions and urea, (c) protons with interfering ions and human serum albumin.

We also characterized the polymer film at different pH values, from 1 to 9, and the results of the measurements are presented in Fig. 8a.

The potential of the film was recorded *vs.* a Ag/AgCl wire reference electrode, and the concentration of each ion in the analyzed solution is specified in the experimental section.

From the linear fit, we could find the slope of the curve, and it was 44.4 ± 0.2 mV per unit pH (*r*^2^ = 0.9976). The Nernstian response slope is 59 mV per decade. The deviation from the ideal value is connected with the fact that we have fluorooctyl side chain groups in our polymer chains. The potentiometric response of the FTO support was recorded and is shown in the ESI in Fig. S4,† because we wanted to prove that the FTO support does not influence the response of the perfluorinated polyaniline films.

The results shown in Fig. 8a are for ideal conditions. However, in real applications, the sensitivity and selectivity must not deteriorate in the presence of interfering ions (in human urine) and/or proteins (blood).

To study the interference of different ions in the response towards H^+^, artificial urine was prepared by the standard procedure in the pH range from 4.0 to 8.5 (the most relevant pH values for human urine),^16^ and the results are shown in Fig. 8b. It is demonstrated that the presence of ions does not decrease the selectivity of the perfluorinated film towards H^+^, and the film also has a Nernstian response with a slope of −39.17 ± 3.18, *r*^2^ = 0.9868 (*n* = 3). When human serum albumin is present in the solution at a concentration of 10 mg mL^−1^, the slope has a value of −41.88 ± 1.8, *r*^2^ = 0.9963 (*n* = 3). Moreover, the response of the perfluorinated film is fully reversible. Therefore, although the absolute value of the working electrode potential is slightly dependent on the concentration of interfering species, the Nernstian response slope is not dependent on the concentration of interfering species, and the concentration of H^+^ ions can thus be reliably measured even in the presence of the abovementioned interfering species typical of the biological milieu. Our results are similar to the reported literature data obtained by other authors.^33–36^

Based on these measurements we conclude that our materials could be used as sensing layers for H^+^.

After the potentiometric measurements, the electrodes were again characterized by high-resolution XPS and the results are presented in the ESI in Fig. S5.† The presence of human serum albumin was detected; however, its attachment does not worsen the performance of the perfluorinated polyaniline films in the detection of H^+^.

## Experimental

### Electrosynthesis

3-Heptadecafluorooctyl aniline (Sigma Aldrich) was polymerized by an electrochemical method; the chronoamperometric method was applied (0.1 mA for 4000 s). Fluorine-doped tin oxide (FTO) glasses with size 2.8 cm^2^ were used as working electrodes, Ag/AgCl (3 M KCl) was used as the reference electrode and Pt wire was used as the counter electrode. Prior to the polymerization, the FTO electrodes were cleaned by using ethanol and then acetone in an ultra-sound bath for 15 min for each solvent. A freshly prepared solution of the monomer was purged with nitrogen gas to avoid the presence of oxygen during the electrochemical synthesis. Ag/AgCl wire was used in the potentiometric measurements. The salts potassium chloride, sodium chloride, sodium hydrogen phosphate and potassium dihydrogen phosphate, and urea were obtained from Sigma Aldrich. Hydrochloric acid (Sigma Aldrich) and sodium hydroxide (Sigma Aldrich) were used to adjust the pH between 4.0 and 8.5.

### Characterization

The water contact angles (WCAs) were measured with an OCA20 (Dataphysics, Germany), and the static CA was determined 10 s after applying a 5 μL water droplet on the polymer film. A minimum of 5 drops were measured.

The surface morphology was analyzed by scanning electron microscopy (SEM). The scanning electron micrographs were obtained using a JEOL 6400 microscope. The transmission electron micrographs were obtained using a Tecnai G2 Spirit (FEI).

The chemical structure was investigated by IR and Raman spectroscopies, and by UV-vis spectroscopy. For infrared spectroscopy, a Thermo Nicolet 6700 FTIR Spectrometer (ThermoScientific, US) with a germanium coated KBr beamsplitter coupled with a Continuum FTIR microscope with a liquid nitrogen cooled MCT/A detector and a Ge tip was used in ATR mode. A Renishaw inVia Reflex Raman microscope (Renishaw, UK) with a high efficiency 250 mm focal length spectrograph equipped with research grade Leica DM LM microscope 785 nm lasers was used. The scattered light was analyzed by the spectrograph with a 1200 lines mm^−1^ holographic grating. UV-vis spectra were recorded on a Perkin-Elmer Lambda 20 UV-vis spectrophotometer.

Electrochemical polymerization was performed on an AUTOLAM potentiostat/galvanostat with an FRA module. All electrochemical measurements (including synthesis) were performed in a three-electrode cell configuration. Polymer deposited on an FTO electrode was used as the working electrode. A Pt wire was used as the counter electrode and Ag/AgCl (3 M KCl) was used as the reference electrode. Nova software 2.11 was used to record the data and all measurements were carried out at room temperature (25 °C).

Electrochemical impedance spectroscopy (EIS) was performed in the frequency range from 10 kHz to 0.1 Hz at open circuit potential (OCP). The Kronig–Kramers test was applied to verify the obtained EIS data. The fitting was performed by using Nova software. The errors associated with each circuit element are:

*R* (0.589%); *C* (6.814%); *R* (4.78%); *Q* (1.12%). The sum of the squares of the relative residuals (*χ*^2^ value) is 0.0195.

Potentiometric measurements at different pH were done *versus* Ag/AgCl wire. An aqueous solution was prepared with 18.2 MΩ cm resistivity deionized water from a Millipore-Q A10 system. Solutions with different pH (from 1.0 to 9.0) were prepared by adjusting the pH with 0.1 M HCl and 0.1 M NaOH aqueous solutions. Artificial urine was prepared according to ref. 16. The interfering species in the artificial urine were urea (9.3 g L^−1^), Cl^−^ (1.87 g L^−1^), Na^+^ (1.17 g L^−1^), and K^+^ (0.75 g L^−1^). To investigate the influence of human serum albumin (HSA) on the potentiometric response of the perfluorinated film, 10 mg mL^−1^ HSA was added to the studied solution (phosphate buffered saline and the pH was adjusted with HCl and/or NaOH).

Matrix-assisted laser-desorption ionization time-of-flight mass spectra (MALDI-TOF MS) were obtained from a Bruker microflex MALDI-TOF spectrometer using *trans*-2-[3-(4-*tert*-butylphenyl)-2-methyl-2-propenylidene]malononitrile as a matrix in negative ionization mode.

X-ray photoelectron spectroscopy (XPS) measurements were carried out with a K-Alpha^+^ spectrometer (ThermoFisher Scientific, East Grinstead, UK). The samples were analyzed using a micro-focused, monochromated Al Kα X-ray source (400 μm spot size) at an angle of incidence of 30° (measured from the surface) and an emission angle normal to the surface. The kinetic energy of the electrons was measured using a 180° hemispherical energy analyzer operated in the constant analyzer energy mode (CAE) at 200 eV and 50 eV pass energy for the survey and high-resolution spectra respectively. Data acquisition and processing were performed using Thermo Advantage software. The XPS spectra were fitted with Voigt profiles obtained by convolving Lorentzian and Gaussian functions. The analyzer transmission function, Scofield sensitivity factors, and effective attenuation lengths (EALs) for photoelectrons were applied for quantification. EALs were calculated using the standard TPP-2M formalism. All spectra were referenced to the C1s peak of hydrocarbons at 285.0 eV. The BE scale was controlled by the well-known positions of the photoelectron C–C, C–H, C–O and C(=O)–O C 1s peaks of polyethylene terephthalate and the Cu 2p, Ag 3d, and Au 4f peaks of metallic Cu, Ag and Au, respectively. The BE uncertainty of the reported measurements and analysis is in the range of ±0.1 eV.

To determine the mass loading of perfluorinated polyaniline on the FTO electrode, a METTLER TOLEDO analytical balance with 1 μg accuracy were used. The determination of the mass loading of polymer was done according to the following steps: a dry FTO electrode (3 × 3 cm^2^) was weighed (*m*_1_), and then it was used for electropolymerization, and after the reaction was completed, the FTO electrode with polymer film was washed several times and dried. The FTO electrode with deposited polymer was weighed again (*m*_2_). The deposited mass was determined by *m*_3_ = *m*_2_ − *m*_1_. The associated error is ±0.5 μg.

## Conclusions

In conclusion, we present for the first time a superhydrophobic conductive polyaniline film deposited by the electrochemical method. Such a film can be used as a solid contact for ion-selective electrodes and as a sensor for the detection of various ions in critical care medicine. The superhydrophobicity of the deposited film could be controlled through the deposition time. With an increase in the deposition time from 2000 s to 4000 s, the WCA increased from 120° to 140°. Moreover, we proved by EIS measurements that the film has a low internal resistance, and the areal capacitance of the film is 0.36 mF cm^−2^. The long-term stability of the film, measured by GCD at 0.02 mA cm^−2^, proves that the perfluorinated film maintains its properties, even after 5000 cycles. Simultaneous possession of a high WCA value and low resistance makes the perfluorinated film a good candidate as a solid contact for ion-selective electrodes.

Potentiometric measurements showed the selective detection of H^+^ by the perfluorinated polyaniline film, even in the presence of interfering ions and/or human serum albumin.

## Author contributions

The manuscript was written through contributions from all authors. All authors have given approval to the final version of the manuscript.

## Conflicts of interest

There are no conflicts to declare.

## Supplementary Material

RA-011-D1RA02325J-s001
