# Supplementary material for: Electrochemical deposition of highly hydrophobic perfluorinated polyaniline film for biosensor applications
Source: RSC Adv. 2021 May 25;11(31):18852–9. doi: 10.1039/d1ra02325j (PMC9033481; doi:10.1039/d1ra02325j)
Supplement: RA-011-D1RA02325J-s001 [file RA-011-D1RA02325J-s001.pdf]

Electronic Supplementary Material (ESI) for RSC Advances.
This journal is © The Royal Society of Chemistry 2021

Supplementary material

Electrochemical Deposition of Highly Hydrophobic Perfluorinated Polyaniline Film for Biosensor Applications

Elena Tomšík,[a] Panagiotis Dallas,[b] Ivana Šeděnková,[a] Jan Svoboda,[a] Martin Hrubý[a]*

[a] Institute of Macromolecular Chemistry AS CR, 162 06 Prague 6, Czech Republic

[b] Institute of Nanoscience and Nanotechnology, NCSR Demokritos, Patriarhou Grigoriou & Neapoleos, 15310, Greece

**tomsik@imc.cas.cz;*

KEYWORDS Superhydrophobicity, sensors, polyaniline, perflurorinated materials.

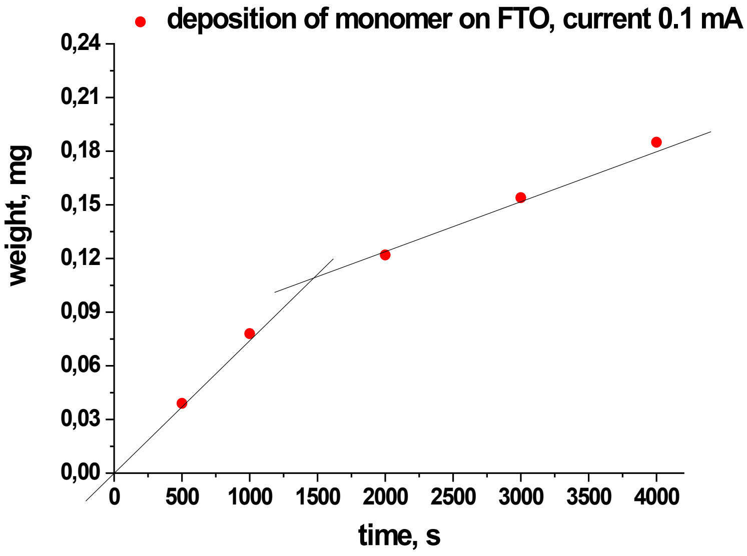

Fig. S1. Dependence of mass loading on duration of deposition.

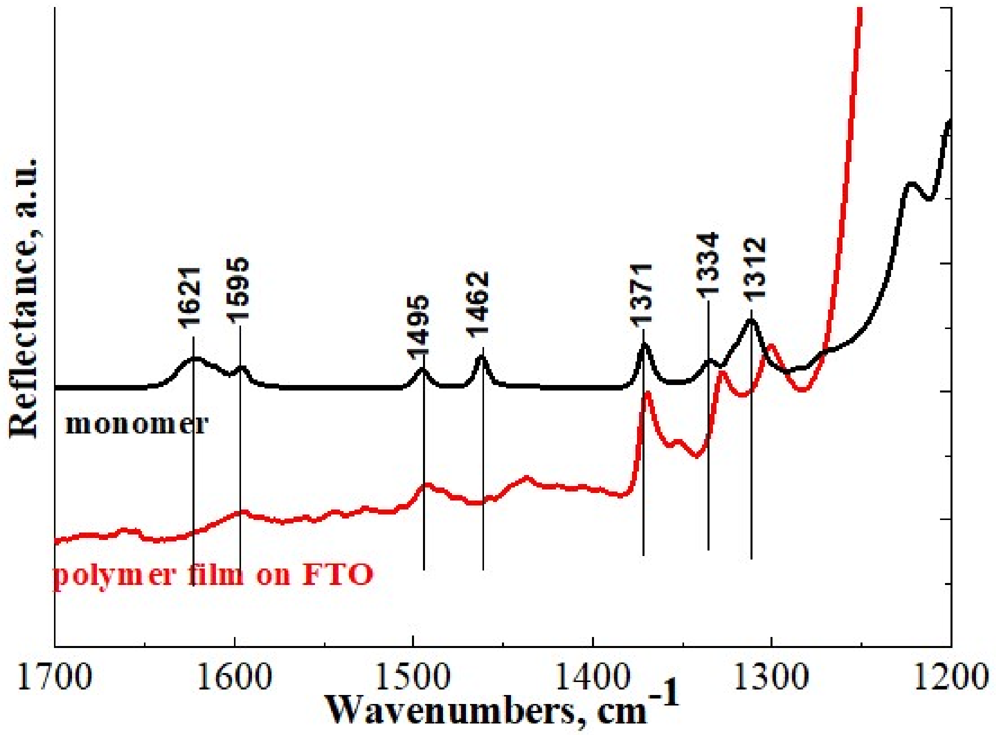

Fig. S2. Raman spectroscopy of monomer and polymer film in the range from 1700 cm^{-1} to 1200 cm^{-1}.

The strong band at 1595 cm^{-1} for polymer corresponds to the C=C stretching in newly formed quinoid rings (the N=Q=N stretching mode).[1,2,3] The band corresponding to C-N stretching vibrations are observed at 1371 cm^{-1} and 1350 cm^{-1}. Kang et al. attributed the 1371 cm^{-1} band in the PANI spectrum to C-N stretching in QB_tQ unit (B_t denotes a trans-benzenoid unit), and the 1350 cm^{-1} band to C-N stretching in QB_cQ (B_c denotes a cis-benzenoid unit). The high intensity of the band at 1371 cm^{-1} provides proves that the delocalized polaronic structure is formed.

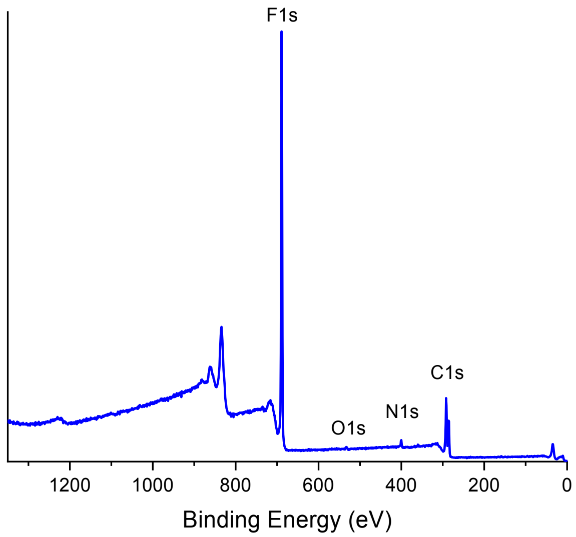

Fig. S3. Survey XPS spectrum of perfluorinated polyaniline film as deposited on FTO electrode.

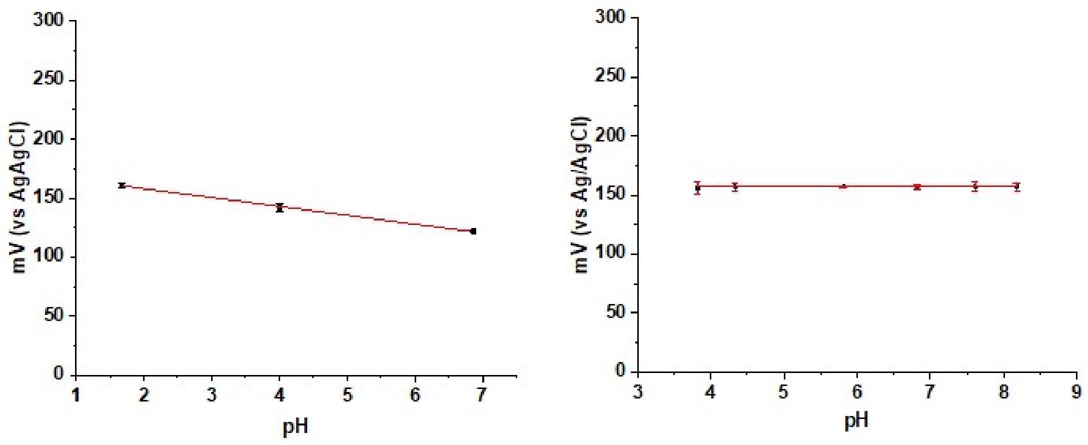

Fig. S4 The potentiometric measurements of pure FTO electrode in solutions with different pH (left) with interfering salts (right).

The measurements were performed for 3 different electrodes. Based on the measured data, we conclude that FTO electrodes could be used as supporting electrode for perfluorinated polyaniline deposition.

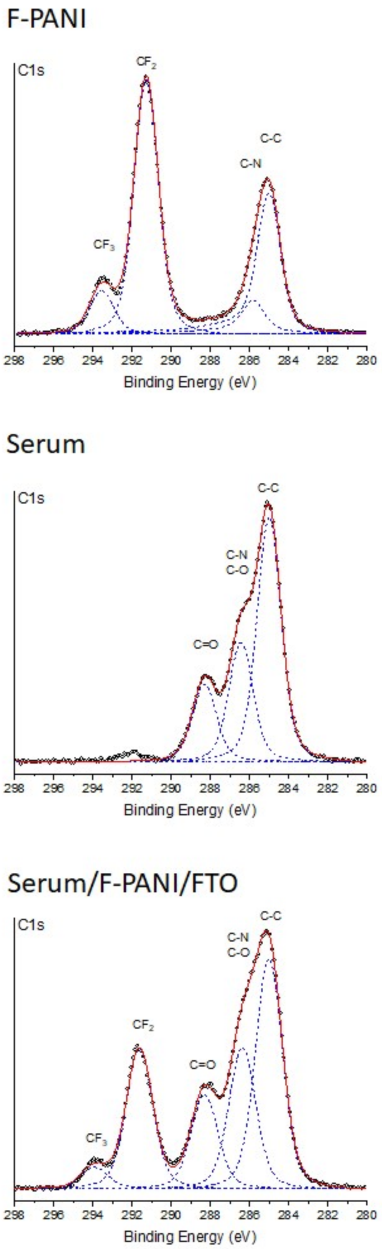

Fig. S5. High-resolution C1s X-ray photoelectron spectra of electrochemically deposited film, and detection of human serum albumin on its surface.

References:

1. Cases, F.; Huerta, F.; Lapuente, R.; Quijada, C.; Morallo´n, E.; Va´zquez, J. L. *J. Electroanal. Chem.* **2002**, 529, 59.
2. Kang, K. G.; Neoh, K. L.; Tan, T. C. *Prog. Polym. Sci.* **1998**, *23*, 277.
3. Kulszewicz-Bajer, I.; Ro´zù alska, I.; Kurylek, M. *New J. Chem.* **2004**, *28*, 669.
